# Isolation and Characterization of Coronavirus and Rotavirus Associated with Calves in Central Part of Oromia, Ethiopia

**DOI:** 10.1155/2020/8869970

**Published:** 2020-11-30

**Authors:** Umer Seid, Fufa Dawo, Asamino Tesfaye, Munera Ahmednur

**Affiliations:** ^1^College of Agriculture, Oda Bultum University, P.O. Box 226, Chiro, Ethiopia; ^2^College of Veterinary Medicine, Addis Ababa University, P.O. Box 34, Bishoftu, Ethiopia; ^3^National Animal Health Diagnostics and Investigation Center, P.O. Box 04, Sebeta, Ethiopia; ^4^Oromia Bureau Livestock and Fishery Resources, West Hararghe Zone, Chiro Wereda, P.O. Box 226, Chiro, Ethiopia

## Abstract

**Background:**

Coronavirus and rotavirus are most commonly associated etiologies for calves' diarrhoea, resulting in loss of productivity and economy of farmers. However, various facets of diarrheal disease caused by coronavirus and rotavirus in calves in Ethiopia are inadequately understood. A cross-sectional study was conducted with the aim of isolation and molecular characterization of coronavirus and rotavirus from calves in the central part of Oromia (Bishoftu, Sebata, Holeta, and Addis Ababa), Ethiopia, from November 2018 to May 2019. The four study areas were purposively selected and faecal samples were collected by simple random sampling for diagnosis of coronavirus and rotavirus infection by using the antigen detection enzyme-linked immunosorbent assay (Ag-ELISA) kit. In addition, this study was carried out to have insight in prevalence and associated risk factors of coronavirus and rotavirus infection in calves.

**Result:**

During the study, 83 diarrheic and 162 nondiarrheic faecal samples collected from calves less than 4 weeks of age were screened for coronavirus and rotavirus. Of the 83 diarrheic samples, 1 sample (1.2%) was positive for coronavirus antigen and 6 samples (7.2%) were found to be positive for rotavirus antigen by Ag-ELISA. All the nondiarrheic samples were negative for both coronavirus and rotavirus Ag. The overall prevalence of coronavirus and rotavirus infection in calves was estimated at 0.4% (1/245) and 2.45% (6/245), respectively. All samples (7) of ELISA test positive of both coronavirus and rotavirus were propagated in Madin-Darby bovine kidney (MDBK) cells. After 3 subsequent passages, progressive cytopathic effect (CPE), i.e., rounding, detachment, and the destruction of monolayer cell of five samples (1 sample of coronavirus and 4 samples of rotavirus) (71.4%) were observed. At the molecular stage, reverse transcriptase polymerase chain reaction (RT-PCR) technique was used to determine the presence of coronavirus and rotavirus nucleic acid by using specific primers. The 5 samples that were coronavirus and rotavirus antigen positive by ELISA and develop CPE on cell culture were also positive on RT-PCR technique. The prevalence of infection peaked at 1st and 2nd weeks of age in male calves.

**Conclusion:**

Diarrheal disease caused by coronavirus and rotavirus has a great health problem in calves that interrupts production benefits with reduced weight gain and increased mortality and its potential for zoonotic spread. So, the present findings show coronavirus and rotavirus infection in calves in Ethiopia that needs to be addressed by practising early colostrum feeding in newborn calves, using vaccine, or improving livestock management.

## 1. Background

Diarrhoea in neonatal calves is one of the most challenging clinical syndromes encountered by the practising large animal veterinarians worldwide. It is caused by multifactorial agents such as viruses, bacteria, and protozoa [1]. Among these etiological agents, coronavirus and rotavirus alone account for about 27–36% [2], resulting as the most common viral enteric pathogens.

Coronaviruses (CoVs) (order *Nidovirales*, family *Coronaviridae*, and subfamily *Coronavirinae*) are enveloped viruses with a positive sense, single-stranded RNA genome. With genome sizes ranging from 26 to 32 kilobases (kb) in length, CoVs have the largest genomes for RNA viruses that are responsible for enteric, respiratory, or neurological signs in mammals and birds. CoVs are classified into 3 groups based on antigenic and genetic properties: *α*-CoVs, *β*-CoVs, and *γ*-CoVs. Bovine coronavirus (BCoV) is included in group *β*-CoVs, which also includes the closely related HCoV-OC43, which causes respiratory infections in humans and the human pathogens SARS-CoV and MERS-CoV [3] and the most recent SARS-CoV-2.

Bovine coronavirus was first identified as the agent of severe diarrhoea in neonatal calves. It was also associated with the occurrence of respiratory distress in calves and adults [4, 5]. The coronaviral genome encodes four major structural proteins: the spike (S) protein, nucleocapsid (N) protein, membrane (M) protein, and envelope (E) protein, all of which are required to produce a structurally complete viral particle [6].

High genetic diversity in coronaviruses is attributable to the high mutation rates associated with RNA replication, the high recombination frequencies within the coronavirus family, and the large coronavirus genomes. Recombination in coronaviruses plays an important role in virus evolution and can result in the emergence of new pathotypes [7]. Thus far, recombination regions in coronaviruses have been extensively reported for the S gene [8]. BCoV is transmitted via the fecal-oral or respiratory route [9]. It infects epithelial cells in the respiratory tract and the intestines, the nasal turbinates, trachea, and lungs, and the villi and crypts of the small and large intestine, respectively. Replication leads to shedding of virus in nasal secretions and faeces. Important factors for the pathogenesis are still not fully explored, such as how the virus infects enterocytes shortly after introduction to an animal. Clinical signs range from none to severe and include fever, respiratory signs, and diarrhoea with or without blood [10].

Rotavirus is a genus of double-stranded RNA virus in the family Reoviridae. The family Reoviridae is composed of eight genera: *Orthoreovirus, Orbivirus, Coltivirus, Rotavirus*, *Aquareovirus, Cypovirus, Phytoreovirus,* and *Fijivirus*. Rotaviruses are characterized by three important antigenic specificities: group, subgroup, and serotype. Group A rotaviruses are major pathogens in humans and animals. The 70 nm diameter wheel-shaped particles consist of a double-layered icosahedral capsid enclosing a core particle that contains 11 segments of double-stranded RNA, each segment representing one gene [11]. Genetic reassortment is one of the important mechanisms for generating genetic diversity of rotaviruses and eventually for viral evolution [12].

Bovine coronavirus and rotavirus are the most recognized pathogens causing acute diarrhoea in cattle and buffalo calves under one month of age worldwide [13]. It has also been recognized as the major pathogen of acute diarrhoea in both humans and animals. So, it has the potential of zoonotic and economic impact [14].

Coronavirus and rotavirus are environmentally distributed worldwide and were highly studied [15, 16]. In a lot of studies, BRoV infection rates of 20–60% in samples of diarrhoea have been reported [17, 18]. Prevalence of coronavirus was estimated ranging from 4% to 38.9% in the sample of diarrheic calves throughout the world. In developing countries, the prevalence of coronavirus and rotavirus was 38.9% and 16.7% [19]. Coronavirus and rotavirus in calves are not well studied in Ethiopia. However, only one report by Abraham et al. [19] indicated the presence of 38.9% coronavirus and 16.7% rotavirus in calves in central Ethiopia. Such lack of the information could be the reason for absence of any strategy for control of coronavirus and rotavirus infection of calves through vaccination by the Ethiopian government. Absence of study conducted on isolation and molecular characterization of coronavirus and rotavirus in calves in Ethiopia may exacerbate the problem. Hence, detecting the circulating strains of coronavirus and rotavirus isolate and molecular evolution of the virus is needed for planning proper control and preventive measure in the country.

Therefore, the objectives of current study were

- To detect coronavirus and rotavirus from calves less than one month of age
- To estimate the prevalence and identify the risk factors of coronavirus and rotavirus infections in calves
- To isolate and characterize the virus by using PCR in the study area

## 2. Methods

### 2.1. Description of the Study Area

**Figure 1:**
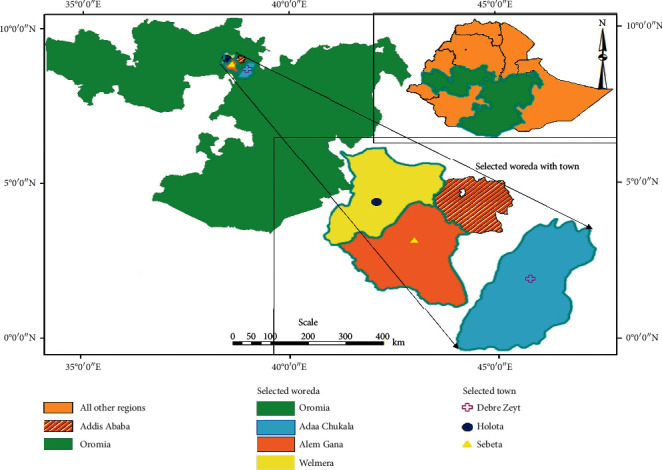
Map of the study area and sampling sites (generated by Umer Seid, corresponding author).

The current study was conducted in four selected areas of the central part of Oromia, Ethiopia (Bishoftu, Sebata, Holeta, and Addis Ababa) from November 2018 to May 2019 (Figure 1). Faecal samples were collected from four selected dairy farms. The dairy farms were representative of many small-, medium-, and large-scale dairy farms in the selected areas that supply milk and milk products to consumers of the neighboring towns and surrounding urban areas. These dairy farms contain either local or exotic breeds depending on the scale of production.

Bishoftu town is found in east Shewa Zone, Oromia Regional State, located about 45 km south-east of the capital city, Addis Ababa. The area is located at 9°N latitude and 40°E longitude at an altitude of 1850 m above sea level. According to national meteorology agency (NMA) (2016), annual rainfall is 866 mm of which 84% is in the long rainy season (June to September) with annual minimum and maximum temperatures of 11 and 29°C, respectively. The domestic animals reared in Bishoftu town are 30887cattle, 43138 poultry, 9322 equine, 9294 sheep, and 4753 goats (Bishoftu City Administration Agricultural Desk, 2014).

Sebeta town is located in the Oromia Special Zone surrounding Finfinne (Addis Ababa) of Oromia Region. The district is located 25 km south west of Addis Ababa at an altitude of 1800–3385 m above sea level and latitude and longitude of 8°55–8.917°N and 38°37–38.617°E, respectively. It receives an average annual rainfall of 1073 ml and has a temperature that ranges from 11.3 to 28°C. It has a total area of 102,758 km [20]. According to the information obtained from Sebeta Hawas district Administration Office [20], both livestock rearing and crop production are the main economic activities of the majority of communities. The major livestock reared in the district includes cattle, sheep, goats, and poultry.

Holeta Town is located in the central part of the country, 31 km west of Addis Ababa in Oromia Regional State, West Shewa Administrative Region. The area is bounded between latitude 8°53′ 75″ to 9°14′ north and longitude 38°21′ 40″ to 38°36′ 14″ east. The town has an area of 5550 hectares. Holeta Town is found at an average 2449 m above sea level. The annual mean maximum and the minimum temperatures are 25.9 and 7.2°C, respectively [21].

Addis Ababa, the capital city of Ethiopia, lies at an elevation of 2300 m above sea level and is featured by a grassland biome. It is geographically located at 9°1′48″N latitude and 38°44′24″E longitude. It has a typical highland climate with temperature ranging from 11°C–24°C. Addis Ababa has a mean annual rainfall of 1300 mm with bimodal distribution [22].

### 2.2. Study Population

The study was conducted in calves both healthy or with clinical signs of diarrhoea, namely, profuse watery diarrhoea, systemic dehydration, and apathy. Cow calves up to 30 days of age including all breeds and sex-reared under intensive management conditions were involved in the study. Diarrhoea was considered if faeces were semiliquid to liquid, with or without other abnormal characteristics such as the presence of blood or mucous. Any calf with faeces without these characteristics was considered nondiarrheic or apparently healthy [23].

### 2.3. Study Design

A cross-sectional study was conducted in different dairy farms found in four selected sites of central part of Ethiopia region of Oromia (Bishuftu, Sebeta, Holeta, and Addis Ababa), from November 2018 up to May 2019. Information about the calves was gathered by interviewing farm owners and animal health workers of selected study sites. The calves were clinically examined for the presence of diarrhoea or not and faecal samples were collected for diagnostic testing. At the time of sampling, the name of the farm, date of sampling, consistency of faeces, age, breed, and tag number were recorded for each calf on the proper recording format.

### 2.4. Sampling Technique and Sample Size Determination

Before the commencement of the actual study, preliminary data were sourced from the respective District Agricultural Office and dairy cooperatives to document the lists of dairy farms into large-scale, medium-scale, and small-scale dairy farms to estimate the size of the study population. The study areas were purposively selected and identified based on transport accessibility and geographical location and on the abundance of dairy farms to get more calves. Clinically diseased and nondiseased calves were sampled for isolation and characterization of rotavirus. The calves from the seven large-scale dairy farms, namely, Genesis Farm, Asterwaqu Dairy Farm, Mama Dairy Farm, Sisay Dimma Dairy Farm, Haddish Dairy Farm, Fantu Dairy Farm, and Holeta Agricultural Research Center Dairy Farm and a representative random sample of calves from 680 medium- and small-scale dairy farms were selected for the study. The sampling units were both local and crossbred dairy calves aged between birth and 1 month. Farms were categorized into small, medium, and large based on the herd size of (5–20), (21–50), and greater than 50 heads of cattle, respectively. In larger farms, a minimum of 10% of the all calves in the farm was sampled.

Considering individual members of dairy cooperatives in each study location as a cluster, cluster sampling method was used to select calves from medium- and small-scale dairy farms. In this study, the sampling frame for study herds was taken from the dairy cooperatives located in Bishuftu, Sebeta, Addis Ababa, and Holeta. A total of 680 medium- and small-scale dairy producers were registered in the dairy cooperatives of study areas. Accordingly, 170 dairy producers were sampled by using a systematic random sampling technique (every 4^th^ dairy producer) from the documented sampling frame. When a selected dairy farmer did not have a calf or no pregnant cows with due calving date in the six-month cohort period, the farmer was then replaced by another dairy farmer mostly from the nearby area. Sample size for cluster sampling was determined by adjusting the sample size calculated for simple random sampling. The adjustment is the function of average cluster size and intracluster correlation and mathematically expressed as follows:

(1) $$\begin{array}{c} n^{\prime }=n\left[\left(1+\left(\left(m-1\right)*\rho \right)\right)\right], \end{array}$$

where *n*′ = sample size for cluster sampling, *n* = sample size calculated for simple random sampling, *m* = average cluster size, and *ρ* = intracluster correlation.

However, in the present study, the average herd (cluster) size (calves per each dairy farm) was 1.6. As clustering was found small, the effect of intracluster correlation would be small and *n*′ would approximate *n*. So, the sample size calculated for random sampling was taken directly to be the sample size for this study. To estimate the prevalence of bovine rotavirus in calves, sample size was determined by using a simple random sampling method [24, 25].

(2) $$\begin{array}{c} n=\frac{1.962\left(p\right)\;\left(1-p\right)}{d^{2}}, \end{array}$$

where *p* = expected prevalence, *d* = desired level of precision (5%), and *d*^2^*n* = sample size.

Using expected rotavirus prevalence 16.7% in central Ethiopia [19], confidence level of 95%, and required absolute precision of 5%, a total of 214 sample sizes was determined for medium- and small-scale dairy farms of the selected study area. However, a total of 245 calves were enrolled during the study period to enhance precision and to compare prevalence across different herd sizes. Among them, 214 calves were from 680 medium- and small-scale dairy farms and 31 calves from seven large-scale dairy farms.

#### 2.4.1. Collection of Faecal Samples

Faecal samples were collected in a sterile tube after cleaning the anal area with a paper towel and beats by rectal stimulation with the index finger using disposable sterile plastic gloves [23]. Approximately, 30 grams of faecal material was collected directly from the rectum of calves using disposable latex gloves. Collected samples were placed into a universal ice box containing ice packs and transported to the virology laboratory at National Animal Health Diagnostic and Investigation Center (NAHDIC), Sebeta, and were stored at −80°C until processing.

### 2.5. Faecal Sample Processing

Two hundred forty-five faecal specimens obtained from diarrheic and nondiarrheic calves were submitted to the National Animal Health Diagnostic and Investigation Center (NAHDIC), Sebeta, from November 2018 to May 2019. Faecal samples were prepared as a 10% (wt/vol) suspension of faeces in 0.01 M phosphate-buffered saline (PBS; pH 7). All samples were centrifuged at 1,500 ×g, and the supernatants were tested and then stored in sterile vials at −80°C for further study.

### 2.6. Laboratory Techniques

#### 2.6.1. Detection of Bovine Coronavirus and Bovine Rotavirus Antigen by ELISA

Multiscreen Ag-ELISA Calf Digestive (BIO K 314/1, Belgium) is a sandwich ELISA capturing mixture of monoclonal antibodies (MAbs) against bovine coronavirus (BCoV) and bovine rotavirus (BRoV). The sandwich ELISA procedure was performed according to the manufacturer instruction.

The optical density was measured at 450 nm using an ELISA plate reader at 450 nm immediately after stopping the reaction with the stop solution.

#### 2.6.2. Extraction of Bovine Coronavirus and Rotavirus RNA

Coronavirus and rotavirus RNAs were extracted from the faecal suspension using QIAamp viral RNA mini kit (Qiagen, Crawley, West Sussex, UK) following the manufacturer's instructions. About 1 g of the faecal sample was added to 1 ml of phosphate buffer saline (PBS). The mixture was vortexed vigorously for 40 seconds followed by centrifugation at 10,000 rpm for 5 minutes. All the supernatant (about 500 *μ*l) was transferred to new tubes. Final extracted viral RNA was stored at −80°C for further processing.

#### 2.6.3. Viral Isolation

All ELISA positive faecal samples were taken forward for virus isolation. Approximately, 1 gram faecal sample was mixed with 9 ml sterile PBS containing antibiotic. The faecal suspension was then centrifuged at 800 rpm for 15 minutes. The supernatant fluids containing coronavirus and rotavirus positive were filtered through 0.45 *μ*m membrane syringe filter, and the filtrates were mixed with an equal volume of Dulbecco's Modified Eagle Medium (DMEM) containing 5% fetal calf serum (FCS) and 10 *μ*g/ml crystalline trypsin and incubated at 37°C for 60 minutes. After incubation, 1 ml of the mixture was inoculated into the culture flasks with confluent monolayer of Madin-Darby bovine kidney (MDBK) cell lines and kept for 1 hour incubation for adsorption of the virus. After the adsorption at 37°C for 1 hour, the cells were washed three times with a plain DMEM maintenance media and incubated at 37°C in a humidified incubator having 5% CO_2_. Monolayers were observed daily for development of CPE for five days and viruses were subcultured blindly every two days after being subjected to 3 cycles of freezing and thawing. CPE was observed after 48 hours and it was characterized by a destruction of the monolayer cell, cell rounding, and infected cells were disrupted and detached from the flask. Cells showing characteristic CPE were harvested by freezing and thawing thrice and centrifuged at 16,000 rpm for 20 minutes at 4°C for the removal of cell debris. The supernatant containing the virus was collected and stored at −80°C for further passages. If no CPE was observed, the sample was considered as “no virus detected” (NVD) and the culture was frozen at −80°C and then thawed and centrifuged at 3,000 rpm for 10 minutes to collect the supernatant for second blind passage (P2). This was repeated for third passage (P3), and if no CPE was observed on the third passage after 48 hours inoculation, then the sample was considered negative for both coronavirus and rotavirus.

#### 2.6.4. Reverse Transcription-Polymerase Chain Reaction (RT-PCR)

The cDNA synthesis was performed with a RT-PCR Kit (QIAGEN), according to manufacturer's instructions, for the confirmation of bovine coronavirus and rotavirus A and random primers in a 25 *μ*L final reaction volume. The cDNA of each sample was screened separately for the BCV and BRV genome using the primers described in Table 1 based on the previous study [26, 27]. PCR reactions were performed according to manufacturer instructions.

Optimized reaction mixture for RT-PCR was dsRNA 2.5 *μ*l, PCR buffer 2.5 *μ*l, dNTPs 2.5 *μ*l, MgC_2_ 2.5 *μ*l, forward primer (10 pmol) 3.0 *μ*l, reverse primer 10 *μ*M (6 *μ*l), and DNase/RNase free water 6 *μ*l. 2.5 *μ*l of viral dsRNA was denatured at 95°C for 5 minutes and chilled immediately for 5 minutes. To analyze the PCR product, agarose gel electrophoresis was performed. For this, 1.5% gel was prepared and 1 *μ*l of 100 base pair (bp) ladder along with the PCR product was run at 110 volts for 45 minutes. The size of the PCR product for gene segment was illuminated in a gel documentation system and a photograph was taken.

### 2.7. Data Management

The collected data were entered in Microsoft Excel. The contingency table was used at 5% significance to assess the differences among the proportions of faecal samples positive to coronavirus and rotavirus variables such as age group and sex of the animals studied by using Chi Square. Quantitative data were coded and entered in a computer spread sheet and the R software was used for the data analysis.

## 3. Results

In total, 245 diarrheic and nondiarrheic faecal samples of calves and their relevant herd and farm level information were collected and analyzed to determine the prevalence of bovine coronavirus and rotavirus infection in dairy calves in the central part of Oromia, Ethiopia. Among 245 calves, 83 (33.88%) had diarrhoea and 162 (66.12%) had no diarrhoea at the time of sampling (Table 2). The overall prevalence of coronavirus and rotavirus infection in calves less than four weeks of age was estimated as 0.4% (1/245) and 2.45% (6/245) by antigen-capture sandwich ELISA in selected dairy farms, respectively (Table 2).

When the results were calculated separately for the two groups of calves (i.e., diarrheic and nondiarrheic calves), a prevalence of 1.2% (1/83) of coronavirus and 7.23% (6/83) of rotavirus was observed in diarrheic calves, and all nondiarrheic samples were negative for both (0/162, Table 2).

Distribution of antigen positive samples corresponding to ages, breed, and sex of calves is shown in Table 2. The results indicate a prevalence for calves in the first and second weeks of ages was 4% and 0.9% for rotavirus and coronavirus, respectively. This shows that new born calves of 1-2 weeks of age were more susceptible to coronavirus and rotavirus infection. But the observed coronavirus and rotavirus prevalence in different calve ages was not significant (*P* > 0.05) (Table 3).

Current research showed that the males were more susceptible to coronavirus and rotavirus infection as compared to female calves. A higher prevalence of 1.2% (1/81) of coronavirus and 6.2% (5/81) of rotavirus was associated with male calves, while a prevalence of 0% (0/163) and 0.6% (1/164) was recorded in female calves, respectively. The prevalence was significant only for rotavirus between male and female calves. A higher prevalence of coronavirus and rotavirus Ag was observed among calves fed colostrum from 30 minutes to 2 hours compared to calves given colostrum within 30 minutes of birth. Newborn calves of exotic breed cows were more susceptible to coronavirus and rotavirus infection than the local breed. The prevalence was not statically significant (*P* > 0.05) for the breed and between times of colostrum feeding. The current study indicated that the prevalence of coronavirus was higher in Addis Ababa (0.8%) and rotavirus infection was higher in Sebeta (4%) as compared with other selected sites. The prevalence was not significant (*P* > 0.05) between the selected locations (Table 3).

Calves' floor area made of concrete is more susceptible to coronavirus and rotavirus infection than the calves' floor area made of brick or mud. The coronavirus and rotavirus prevalence was not significant (*P* > 0.05) between the calves' floor areas. The result indicated that 6.9% calves separated immediately after birth from dam were found positive for rotavirus, whereas coronavirus and rotavirus were detected in 0.7% and 2.6% samples of calves separated greater than 24 hours after birth from dam, respectively (Table 2). Prevalence values were not statically significant (*P* > 0.05) for coronavirus and rotavirus between the times of separations of calves from dam (Table 3). See Table 2 for stratified prevalence of other variables.

In this study, MDBK cell line was used to isolate the virus from all samples of Ag-ELISA test positive samples. Out of 7 samples of both coronavirus and rotavirus cultured on MDBK cell line, CPE was observed in 5 (1 coronavirus and 4 rotavirus) samples, while CPE was not observed on the remaining 2 samples of rotavirus even on third blind passage. In the first passage, infected cells did not show any CPE. But from the second passage onwards the infected cells started showing characteristic CPE. At 24 hours after infection (postinfection (p.i.)), the infected cells became round and clumped. At 48 hours p.i., the cells were thin and round shaped. At 72 hours p.i., the cells became small and the majority of monolayer was detached.

Out of 7 positive samples, only 5 (1 sample of coronavirus and 4 samples of rotavirus) samples were screened by RT-PCR for molecular characterization due to the nonavailability of sufficient quantity of faecal samples in the remaining samples. Out of the 5 (1 sample of coronavirus and 4 samples of rotavirus) faecal samples of coronavirus and rotavirus examined by RT-PCR technique, all samples were identified as positive (100%) for RT-PCR test.

## 4. Discussion

Newly born calves are an important source in livestock production worldwide for meat or breeding, i.e., replacement stock [28]. This industry faces many disease problems such as calf diarrhoea, which usually affects it dramatically. Calf diarrhoea is a prime disease affecting newborn calves leading to morbidity and mortality in newborn calves, causing economic losses due to the costs of treatment, diagnostics, weight loss, or death in infected animals and poor growth performance. A crucial period for these calves is the first few days following birth. In developing countries such as Ethiopia, domestic animals are the major income source for poor families. These families suffered badly due to the neonatal calf mortality curse. Among numerous viral, bacterial, and parasitic causative agents, bovine coronavirus and rotavirus is the foremost cause of neonatal calf diarrhoea in domestic animals. The cause of neonatal calf mortality is specifically related to bovine coronavirus and rotaviruses [29]. Faecal contamination plays an important role in the transmission of coronavirus and rotavirus infection and the infections are widespread globally in cattle populations. For the effective control measures, prompt diagnosis of the disease is important [30].

In the present study, 1 of 245 (0.4%) and 6 of 245 (2.4%) faecal samples screened using Ag-ELISA were positive for coronavirus and rotaviral infection, respectively; all nondiarrheic samples were negative for both (0/162).

Out of the 83 diarrheic samples, 1 (1.2%) was found positive for coronavirus by ELISA. Other studies also revealed that prevalence of corona virus in neonatal calf diarrhoea is slightly lower than that of rotavirus. However, there is paucity of literature stating the corona virus prevalence status in Ethiopia. This study is in agreement with the report by Dash et al. [31] in India (4.76%). Most of the other reports disagreed with the current study.

A prevalence of 7.23% (6/83) of rotavirus was observed in diarrheic calves in the current study. This result is in agreement with those reported by Pérez et al. [32] in Costa Rica (7%), Yilmaz [33] in Turkey (8.92%), and Rajendran and Kang [34] in India (5.5%). Higher prevalence rate of rotavirus has been reported by authors from many countries including Abraham et al. [19] in Ethiopia (16.7%), Ammar et al. [23] in Algeria (14.63%), Kyle [35] in Vietnam (15%), Pisanelli et al. [36] in Southern Italy (16.8%), Jindal et al. [37] in India (27.02%), and Uhde et al. [18] in Switzerland (58.7%). However, the result of this study is higher when compared to that reported by Fiedler et al. [38] in Oldenburg (1.96%). The discrepancy of results could be attributed to the age and the sample size difference. Prevalence of rotaviral infection varies depending on the country and region under study [28, 39]. All the 6 rotavirus positive samples were from diarrheic calves under the age of 4 weeks. Similar results were recorded by Sharma [40] in bovine calves.

The current result could suggest that male calves (6.2%) were highly susceptible to rotavirus infection than female calves (0.6%). Other studies by Dash et al. [31] and Sharma [40] also reported higher susceptibility of male bovine calves (20.37% and 42.85%) in comparison to female calves (12.76% and 28.2%), respectively. The possible justification for this could be due to immune system in that Odde [41] reported higher antirotavirus IgG concentrations for female calves compared to male calves. It could be due to the managemental practices, as in most of the dairy farms, female calves are better looked after than male calves. Previously, Ammar et al. [23] and Dash et al. [31] also reported higher susceptibility of male bovine calves in comparison to female calves against rotavirus infection. In line with this, others noticed that male calves were more susceptible to diarrhoea as compared to female calves.

Age-wise, the susceptibility of newborn calves of 1^st^ week up to 2^nd^ week of age to rotavirus infection was more than older calves. The occurrence of rotavirus in the faecal samples of diarrheic calves was found to decrease with increase in the age of the calves. The finding of the present study is in agreement with the earlier workers reported by Abraham et al. [19]; higher occurrence of rotavirus infection in diarrheic calves was mainly restricted to the first 2 weeks of life. Maximum prevalence of rotavirus diarrhoea was observed in 5–21-day-old calves [28]. The 2-week-old calves were most susceptible to rotavirus infections, which may be due to decreasing of passive immunity and the absence of the natural resistance against infection. The 3-week-old calves are characterized by the absence of rotavirus; this may be highlighted by an increased natural resistance against infection [23].

The results showed that the prevalence was slightly higher in the Sebeta (4%) than in Bishoftu (3.3%) and Addis Ababa (2.4%) towns. This could be attributed to the sample size of the areas and the presence of a higher number of factories in the near farm that can be a source of contamination for animals. In the present study, higher prevalence was recorded more in crossbred calves (4.2%) than in local calves (2.8%) that is similar to the previous report by Sharma [42].

In the present study, viral growth in cell culture was assessed by examining inoculated cells for CPE. Out of 7 samples, only 5 (1 sample of coronavirus and 4 samples of rotavirus) of the Ag-ELISA positive samples established infection in MDBK cells as determined by the production of characteristic CPE on the second passage and it continued up to third passage. The CPE observed was characterized by rounding, detachment, as well as the destruction of the monolayer cell. The CPE produced in this study agreed with the previous reports [43].

The RT-PCR technique confirmed the presence of coronavirus and rotaviruses in faecal samples that were previously diagnosed by ELISA and growth in cell culture. The RT-PCR-based genotyping method used was further confirmed to be a useful epidemiological tool and to determine the presence of coronavirus and rotavirus nucleic acid by using specific generic primers for each virus regions in faeces samples [44].

## 5. Conclusion and Recommendations

The present investigation was undertaken to investigate the prevalence, isolation, and characterization of coronavirus and rotavirus in calves less than one month of age at different selected farms in central Oromia. The effect of age, sex, breed, and house floor of calves on the prevalence of diarrhoea was also studied. Using Ag-ELISA, 7 samples (1 coronavirus and 6 rotavirus) were identified as positive, and all of the isolates were obtained from diarrheic calves in the 1^st^ and 2^nd^ weeks of age. The result indicated that there is an association between rotavirus detection and sex of calves in that the prevalence of rotavirus is higher in male calves than female calves. In addition, the prevalence is higher in calves kept in concrete floor, this fed colostrum later (within 2 hours) as well as in local bred calves and in calves separated from their dams immediately after birth.

Observational study and questionnaire survey also indicated that not only awareness of the advantage of colostrum feeding is enough but also times of colostrum administration to neonate calves are crucial for the ultimate development of immune status against pathogens including coronavirus and rotavirus infection. Calving areas should have well-drained grass lots or pastures visible from the barn area, and calving areas should be selected or landscaped to allow for adequate drainage. Enteric disease such as coronavirus and rotavirus infection is a vital health problem in calves that interrupt production benefits with reduced weight gain and increased mortality, and the virus potential for its zoonotic spread, it is imperative to determine the disease burden and responsible risk factors. This is very useful to execute effective preventive measures such as practicing early colostrum feeding in newborn calves, vaccination in dams, and improving livestock management.

Based on the above conclusion, the following recommendations were forwarded:

- Awareness creation for researchers and the government regarding the effect of coronavirus and rotavirus infection in calves' health and growth performance and national economy are very important.
- Further study of coronavirus and rotavirus infection in calves covering larger areas of the country needs to be conducted so that representative information of the circulating strains could be generated and understood. Availability of such data is critical for control of the disease.

## Figures and Tables

**Table 1:** Primer details.

Primer	Primer sequences	Size of amplicons (bp)	Melting temp.	Primer length
VP4-F	TGGCTTCGCTCATTTATAGACA	880	54.2	22
VP4-R	ATTTCGGACCATTTATAACC		47.4	20
BCV-F	GCCGATCAGTCCGACCAATC	407	55	20
BCV-R	AGAATGTCAGCCGGGGTAT		52	19

**Table 2:** Frequency distribution of bovine coronavirus (BCoR) and bovine rotavirus (BRoV).

Factors	Level	BCoV			BRoV	
		*N*	−ve	+ve	−ve	+ve
Location	Bishoftu	60	60	0 (0%)	58	2 (3.3%)
	Addis Ababa	123	122	1 (0.8%)	120	3 (2.4%)
	Sebeta	25	25	0 (0%)	24	1 (4%)
	Holeta	37	37	0 (0%)	37	0 (0%)

Clinical status	Diarrheic	83	82	1 (1.2%)	77	6 (7.2%)
	Nondiarrheic	162	162	0 (0%)	162	0 (0%)

Breed	Cross	24	24	0 (0%)	23	1 (4.2%)
	Local	72	72	0 (0%)	70	2 (2.8%)
	Exotic	149	148	1 (0.7%)	146	3 (2%)

Sex	Male	81	80	1 (1.2%)	76	5 (6.2%)
	Female	164	164	0 (0%)	163	1 (0.6)

Age	1^st^ week	100	100	0 (0%)	96	4 (4%)
	2^nd^ week	106	105	1 (0.9%)	104	2 (1.9%)
	3^rd^ week	18	18	0 (0%)	18	0 (0%)
	4^th^ week	21	21	0 (0%)	21	0 (0%)

Floor of the calves' area	Concrete	181	180	1 (0.6%)	177	4 (2.2%)
	Brick	58	58	0 (0%)	56	2 (3.4%)
	Muddy	6	6	0 (0%)	6	0 (0%)

First-time colostrum feeding after birth	Within 30 minutes	132	132	0 (0%)	131	1 (0.8%)
	Within 2 hours	103	103	1 (1%)	98	5 (4.9%)
	Within 2–6 hours	10	10	0 (0%)	10	0 (0%)

Separation of calves from dam	Immediately after birth	29	29	0 (0%)	27	2 (6.9%)
	<24 hours	71	71	0 (0%)	71	0 (0%)
	>24 hours	145	144	1 (0.7%)	141	4 (2.8%)

Total		**245** ^*∗*^	**244** ^*∗*^	**1 (0.4%)** ^*∗*^	**239** ^*∗*^	**6 (2.4%)** ^*∗*^

BCoV, bovine coronavirus; BRoV, bovine rotavirus; *N*, number; −ve, negative; +ve, positive; (%) represents the percentage of the total number of cases; ^*∗*^ indicates the total of each parameter.

**Table 3:** Evaluation of the association between BRoV, diarrhoea, and other variables (age, breed, sex, location, clinical status, means of offering, and separation from dam).

	OR	95% CI	*P* value
(Intercept)	0.000	2.866*e* + 112	0.9945
Age	0.346	0.00341 1.933	0.2766
Breed	1.745	0.0298 14.42	0.5594
Location	0.6751	0.239 1.824	0.4238
Clinical status	6.908*e* + 08	0.000	0.9947
Sex	0.028	0.0004 0.392	0.0358^*∗*^
First colostrum	7.470	1.001 94.70	0.0691
Separate dam	0.3067	0.042 1.596	0.1914
Floor area dam	1.401	0.128 9.936	0.7404

Note: *P* value, level of significance. Significant when *P* value ≤0.05. OR, odd ratio; 95% CI, 95% confidence interval.

## Data Availability

The data used to support the findings of this study are available from the corresponding author upon request.

## Abbreviations

Ag: Antigen
Ag-ELISA: Antigen-capturing enzyme-linked immunosorbent assay
BCoV: Bovine corona virus
BRoV: Bovine rotavirus
cDNA: Complementary DNA
CPE: Cytopathic effect
DMEM: Dulbecco's Modified Eagle Medium
DNA: Dioxyribonucleic acid
DNTPs: Dioxynucleotide triphosphates
dsRNA: Double-stranded RNA
ELISA: Enzyme-linked immunosorbent assay
MDBK: Madin-Darby bovine kidney
mRNA: Messenger RNA
NAHDIC: National Animal Health Diagnostic and Investigation Center
NSP: Nonstructural proteins
NVD: No virus detected
OD: Optical density
p.i: Postinfection
PBS: Phosphate-buffered saline
PCR: Polymerase chain reaction
pH: Power of hydrogen
RNA: Ribonucleic acid
rpm: Revolution per minute
RT-PCR: Reverse transcriptase-polymerase chain reaction
SHFDO: Sebeta Hawas Finance and Economic Development Office
UK: United Kingdom
VP: Structural proteins.
